# Protective role of acetylcholine and the cholinergic system in the injured heart

**DOI:** 10.1016/j.isci.2024.110726

**Published:** 2024-08-14

**Authors:** Clara Liu Chung Ming, Xiaowei Wang, Carmine Gentile

**Affiliations:** 1 School of Biomedical Engineering, Faculty of Engineering and Information Technology, University of Technology Sydney, Sydney, NSW, Australia; 2 Cardiovascular Regeneration Group, Heart Research Institute, Newtown, NSW 2042, Australia; 3 Department of Medicine, Monash University, Melbourne, VIC 3800, Australia; 4 Department of Cardiometabolic Health, University of Melbourne, Melbourne, VIC 3010, Australia; 5 Molecular Imaging and Theranostics Laboratory, Baker Heart and Diabetes Institute, Melbourne, VIC 3004, Australia.

**Keywords:** Cardiovascular medicine, Pathophysiology

## Abstract

This review explores the roles of the cholinergic system in the heart, comprising the neuronal and non-neuronal cholinergic systems. Both systems are essential for maintaining cardiac homeostasis by regulating the release of acetylcholine (ACh). A reduction in ACh release is associated with the early onset of cardiovascular diseases (CVDs), and increasing evidence supports the protective roles of ACh against CVD. We address the challenges and limitations of current strategies to elevate ACh levels, including vagus nerve stimulation and pharmacological interventions such as cholinesterase inhibitors. Additionally, we introduce alternative strategies to increase ACh in the heart, such as stem cell therapy, gene therapy, microRNAs, and nanoparticle drug delivery methods. These findings offer new insights into advanced treatments for regenerating the injured human heart.

## Graphical abstract

**Figure:**
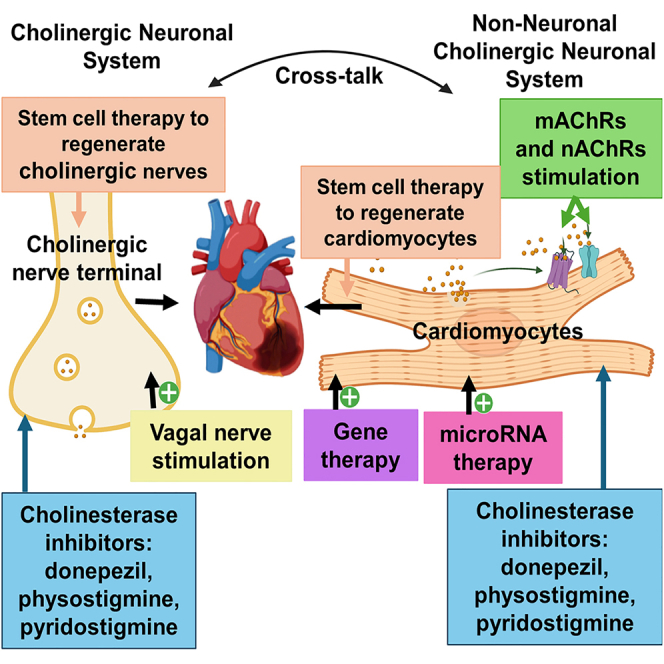

## Introduction

Cardiovascular diseases (CVDs) are a leading contributor to global mortality and morbidity, responsible for an estimated 17.9 million deaths each year, as reported by the World Health Organization.^1^ In the early phases of CVD, an imbalance occurs within the autonomic system, marked by an increase in sympathetic activity and a decrease in the parasympathetic system. This disequilibrium is associated with an increase in norepinephrine and a reduction in the release of acetylcholine (ACh), contributing to higher rates of cardiac mortality and hindering myocardial regeneration.^2^^,^^3^^,^^4^ Early studies reported that an increase in sympathetic activation and parasympathetic inhibition in dogs leads to tachycardia-induced heart failure (HF) .^5^ Additionally, Mahmoud et al.^2^ demonstrated that inhibition of the cholinergic nerve during cardiac injury in neonatal mice and zebrafish leads to incomplete heart regeneration, causing a significant decrease in neonatal cardiac cell proliferation. In clinical trials, such as the Autonomic Tone and Reflexes After Myocardial Infarction study and Cardiac Insufficiency Bisoprolol Study II, reduced cardiac vagal activity is associated with increased heart rate and higher mortality rates in HF patients.^6^^,^^7^ In pre-clinical studies, Guimarães^8^ demonstrated that mice with a long-term cholinergic deficit exhibit increased norepinephrine levels and heightened sympathetic activity, resulting in cardiotoxicity. However, the correlation between CVD and autonomic dysfunction is still not well understood.

Several studies have reported that a decrease in ACh release, stemming from reduced cholinergic activity, is linked with various CVDs, including arrhythmias,^9^ atherosclerosis,^10^^,^^11^ myocardial infarction (MI),^12^^,^^13^ ischemic-reperfusion injury (I/R),^14^^,^^15^ doxorubicin (DOX)-induced cardiotoxicity,^16^ and HF.^17^ Additionally, increasing ACh secretion restores the imbalance of the autonomic system, improves heart rate viability, regulates mitochondrial function, suppresses reactive oxygen species (ROS) production, and alleviates inflammatory responses.^8^^,^^18^ Several studies have shown that the cholinergic system in the heart plays a pivotal role in guiding cardiac regeneration in the injured heart in mice, rats, rabbits, swine, and canine.^19^^,^^20^^,^^21^^,^^22^^,^^23^ Nonetheless, these pre-clinical findings have not been successfully translated to clinical studies, potentially due to the variability in ACh delivery methods across studies and, more broadly, to the multitarget effects of ACh in the human body. Therefore, a better understanding of the potential protective roles of ACh against myocardial injury, as well as the mechanisms regulating this process, will facilitate the development of new therapeutic targets against cardiac damage.

In this review, we focus on both the neuronal and cardiomyocyte cholinergic systems in the heart. Both systems are interconnected and essential for regulating the release of ACh, which binds to muscarinic and/or nicotinic ACh receptors to activate various signaling pathways, thereby maintaining cardiac homeostasis. We discuss the role of ACh derived from both systems in the heart and its therapeutic potential through muscarinic and/or nicotinic ACh receptors against CVD. The review also examines the limitations and constraints of current methods used to increase ACh levels in pre-clinical and clinical trials, such as vagus nerve stimulation (VNS) and cholinesterase inhibitors. Finally, we highlight alternative approaches to target the cholinergic system for more targeted and less invasive therapeutic strategies.

## The cholinergic system of the heart

The autonomic system is composed of the sympathetic and parasympathetic nervous systems, which work in opposition to each other to balance heart activity. The parasympathetic nervous system, also known as the neuronal cholinergic system, encompasses molecules responsible for the synthesis, storage, release, signaling, and degradation of ACh, collectively regulating extracellular ACh concentrations within the presynaptic terminal.^18^ As a neurotransmitter, ACh is employed to modulate cardiac activity through muscarinic ACh receptors (mAChRs) to regulate heart dynamics^8^ and through nicotinic ACh receptors (nAChRs) to modulate inflammatory pathways and cardiac hemodynamic.^24^

**Figure 1:**
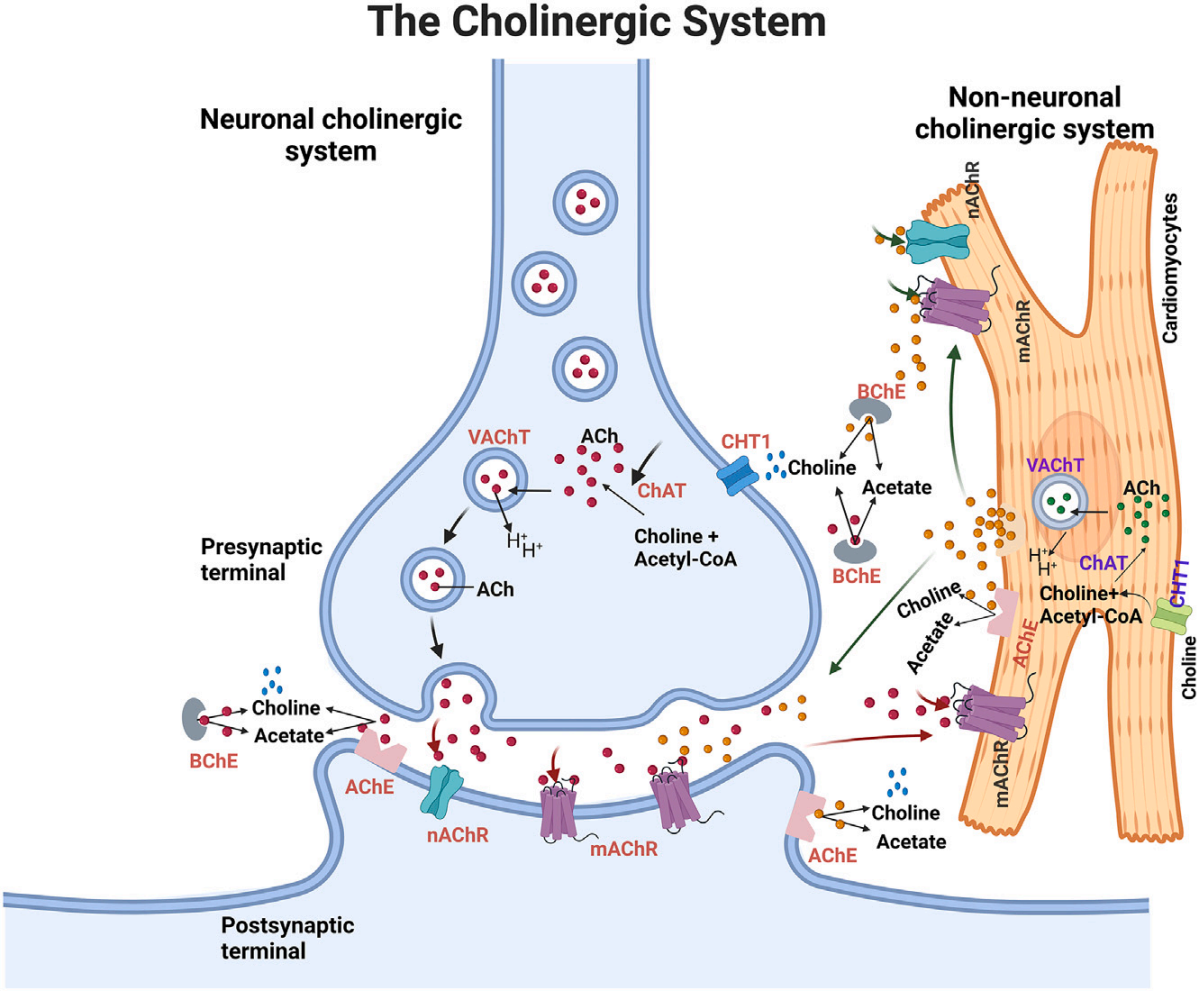
Schematic illustration of the cardiac neuronal cholinergic system in both cardiomyocytes and cholinergic nerves Acetylcholine (ACh) (red) derived from the presynaptic terminal and ACh (orange) derived from cardiomyocytes are synthesized by choline acetyltransferase (ChAT) from the reaction between acetyl-CoA and choline. ACh from the neuronal and non-neuronal cholinergic system is stored by the vesicular acetylcholine transporter (VAChT) and released upon stimulation. ACh released from both systems binds to ligand-gated ion channels nicotinic or G protein-coupled muscarinic ACh receptors (nAChRs and mAChRs, respectively). Acetylcholinesterase (AChE) present on postsynaptic terminal and cardiomyocytes and butyrylcholinesterase (BChE) present in the extracellular space degrade ACh to choline and acetate. High-affinity choline transporters (CHT1), present in cholinergic neurons and cardiomyocytes, are responsible for the reuptake of free choline for ACh synthesis.

As outlined in Figure 1, ACh synthesis is catalyzed by choline acetyltransferase (ChAT), leading to its release and subsequent action in the cardiac extracellular space. The latter combines choline, supplied by a high-affinity choline transporter (CHT1), with acetyl-coenzyme A (CoA).^18^^,^^25^ ACh is stored within synaptic vesicles, which are acidified via an energy-dependent pump (H-ATPase) and mediated via vesicular ACh transporter (VAChT).^26^ Upon depolarization, calcium influx triggers exocytosis, wherein ACh-filled synaptic vesicles fuse with the cellular membrane, releasing ACh in the synaptic cleft.^4^ Once released into the extracellular space, neuronal-derived ACh binds to mAChRs and nAChRs to activate various signaling pathways in the cardiovascular system.^27^^,^^28^^,^^29^ The degradation of ACh occurs rapidly by cholinesterases, including acetylcholinesterase (AChE) and butyrylcholinesterase (BChE). Both AChE and BChE hydrolyze ACh into choline and acetate.^30^ CHT1 then transports choline to the presynaptic terminal.^4^^,^^31^

Over the past two decades, a few studies have supported an additional intrinsic cholinergic machinery in heart cells, also known as the non-neuronal cholinergic system (NNCS) (which includes cardiomyocytes,^14^ endothelial cells,^32^ and leukocytes^32^^,^^33^). Recently, Tarnawski et al.^32^ showed that ACh-derived T cells regulate vascular endothelial function and blood pressure via promoting endothelial nitric oxide synthase activity, vasorelaxation, and reducing vascular endothelial activation. Although leukocytes and endothelial cells have been shown to have the components of cholinergic machinery, their contribution to the total pool of ACh in the heart is low and remains unexplored.^4^^,^^34^

In this review, we also focus on cardiomyocyte cholinergic machinery and the role played by cardiomyocyte-derived ACh in the heart. Cardiomyocytes can synthesize, transport, and release ACh, as they contain enzymes and transporters for ACh synthesis (ChAT), storage (VAChT), degradation (AChE and BChE), and reuptake of choline for synthesis (CHT1) (Figure 1).^35^^,^^36^ Roy et al.^37^ suggested that cardiomyocyte-derived ACh functions through similar second messenger systems and binds to mAChRs or nAChRs similar to neuronal-derived ACh. Additionally, studies have found that cardiomyocyte-derived ACh was detected intracellularly and extracellularly using cholinesterase inhibitors such as donepezil, pyridostigmine, and physostigmine.^35^^,^^36^ Hence, the NNCS in cardiomyocytes comprises those components that regulate ACh homeostasis and act in an auto-/paracrine manner to mediate signaling.^18^ This concludes that both the neuronal cholinergic system and NNCS crosstalk with each other to mediate and regulate homeostasis heart activity.

## The role of cardiomyocyte-derived ACh in the heart

While both the neuronal cholinergic system and NNCS operate concurrently to regulate cardiac homeostasis, cardiomyocyte-derived ACh plays a crucial role in the heart and offers protection against CVD. ACh derived from cardiac NNCS sustains or enhances neuronal cholinergic effects, regulates heart rate, counteracts hypertrophic signals, maintains action potential propagation, and regulates cardiac energy metabolism.^26^^,^^35^^,^^37^^,^^38^^,^^39^^,^^40^ For instance, Roy et al.^40^ found that lowering the levels of cardiomyocyte-derived ACh using ChAT and VAChT knockout mice models resulted in increased heart rate and cardiovascular dysfunction. This suggests that a reduction in cardiomyocyte-derived ACh levels could potentially cause long-term changes in heart function, including ventricular cardiomyocyte hypertrophy, cardiac remodeling, and an increase in ROS.^40^ Similar to neuronal-derived ACh, cardiomyocyte-derived ACh is crucial for maintaining cardiac homeostasis and regulating critical signaling pathways to maintain normal heart activity. Furthermore, Kakinuma et al.^41^ demonstrated that ChAT deletion in murine atrial myocardial cells increased oxygen consumption, mitochondrial activity, and reduction of ATP levels. As a result, cardiac NNCS plays a protective role in cardiomyocytes and the entire heart by maintaining physiological ATP levels and inhibiting oxygen consumption.^42^

NNCS is also crucial for maintaining the balance between parasympathetic and sympathetic heart innervation and could amplify the protective effects of the parasympathetic nervous system.^4^^,^^43^ Few studies have shown that cardiomyocyte-derived ACh could modulate the central nervous system via the afferent vagal nerve, initiating crosstalk with other organs such as the brain, liver, and others.^44^ Increasing cardiac NNCS signaling through a genetic approach and/or pyridostigmine, a cholinesterase inhibitor, leads to protective immunomodulatory effects, such as a reduction in CCL2/7 chemokines expression and a decrease of pro-inflammatory CCR2+ monocytes in the heart following cardiac injury.^45^ This supports the cardioprotective role of cardiomyocyte-derived ACh via the modulation of the innate immune system.

Despite the fact that cardiac NNCS could be a potential target for therapeutic intervention, the detailed mechanisms that trigger the release of cardiomyocyte-derived ACh are still unclear. While Rocha-Resende et al.^26^ proposed that adrenergic stimulation can induce cholinergic gene expression in cardiomyocytes, Roy et al.^37^ suggested that cardiomyocyte-derived ACh may be regulated by sympathetic activity and the intrinsic cholinergic machinery, to regulate heart rate after stress and exercise. However, further studies are needed to identify the factors that activate NNCS in cardiomyocytes.

### The role of cardiomyocyte-derived ACh against CVD

In this sub-section, we explore the potential therapeutic role of targeting cardiac NNCS and the protective role of cardiomyocyte-derived ACh against CVD. The current methods to increase cardiac NNCS activity are cholinesterase inhibitors, including pyridostigmine^26^^,^^45^ and donepezil,^35^ small interfering RNA targeting AChE to increase ACh level,^26^ and overexpression of ChAT gene^42^^,^^46^ or VAChT gene.^45^ Numerous studies have demonstrated that cardiac NNCS reduces oxygen demand and improves oxygen supply during ischemic injury, thereby preventing ischemia-induced cardiac dysfunction^42^^,^^45^ as well as preventing type 1 diabetes-induced heart diseases.^47^

To elucidate the cardioprotective function of the NNCS in ischemic heart disease, Kakinuma et al.^42^ generated a heart-specific ChAT transgenic (ChAT-tg) mouse model that overexpresses ChAT in the heart to increase ACh synthesis. The findings revealed that cardiomyocyte-derived ACh plays an evident role in regulating myocardial energy metabolism through the activation of myocardial glucose utilization and angiogenesis in the infarcted area. Additionally, after 14 days, ChAT-tg mice exhibited increased resistance to MI and a higher survival rate compared to wild-type mice. This could be linked to the fact that cardiomyocyte-derived ACh activates hypoxia-inducible factor (HIF)-1α, a non-hypoxic mechanism, and efficiently prevents energy depletion in the heart.^48^

Moreover, cardiomyocyte-derived ACh increases cellular ATP levels. By monitoring ATP levels in real time, Oikawa et al.^49^ demonstrated that insulin growth factor (IGF)-1R and Glut-1 protein expressions were upregulated together with an increase of ACh-derived cardiomyocytes, leading to a rise in glucose uptake and utilization. This mechanism preserves cellular ATP levels during oxidative stress and suppresses ROS production.^14^ Therefore, overexpression of cardiomyocyte ChAT activates cardiac ACh-HIF-1α cascade and improves cell survival after myocardial I/R. Moreover, Kakinuma et al.^42^ found that upregulation of cardiomyocyte ChAT activates the cardiac ACh-HIF-1α cascade to improve cells in ChAT-tg myocardial I/R mice. Hence, loss of cardiac ACh-HIF-1α transcriptional pathway leads to cardiac dysfunction, which could be due to the synergistic effect of hypovascularity, calcium mishandling, and decreased myocardial energy.^14^

While the increase of sympathetic activity and norepinephrine is associated with arrhythmogenesis, cardiotoxicity, and impaired parasympathetic function, no link has been established between cardiomyocyte-derived ACh and cardiac norepinephrine levels.^8^ Consequently, the specific molecular mechanisms by which cardiomyocyte-derived ACh influences the cardiac cholinergic system remain unknown, underscoring the need for additional research. For instance, Kakinuma et al.^35^ suggested that cardiomyocytes increase the transcriptional activity of the ChAT gene through mAChRs and ChAT protein expression to increase ACh levels in the cholinergic system. Furthermore, studies have shown that the NNCS enhances vagus nerve activity by increasing the release of nitric oxide from cardiomyocytes, contributing to beneficial cardiac effects^42^^,^^44^ as well as extracardiac effect.^50^ Heart-specific ChAT-tg mice play a protective role in the central nervous system via the activation of the vagus nerve.^44^ This pathway is important in the regulation of the inflammatory response in the blood-brain barrier (BBB), as well as in the response to restraint stress, less depressive-like and anxiety-like behaviors, and anti-convulsive effects.^44^^,^^50^ Therefore, it is crucial to conduct further investigations to fully understand the true clinical potential of targeting the cardiac NNCS and its effects on peripheral organs. Future research should also focus on determining the optimal strategies for enhancing NNCS-derived ACh levels in the heart.

## Cardioprotective roles of ACh via mAChRs and nAChRs

Impairments in ACh signaling from both neuronal and NNCS sources can result in cellular death, heart dysfunction, and suppression of anti-inflammatory pathways, mediated by mAChRs and nAChRs.^51^^,^^52^ Stimulation of mAChRs, which are G protein-coupled receptors, leads to a decrease in heart rate and reduced cardiac conductivity.^53^^,^^54^ mAChRs regulate ventricular function both directly by counteracting β-adrenergic stimulation and indirectly by inhibiting L-type calcium channels.^55^^,^^56^ nAChRs are cholinergic ligand-gated ion channels permeable to Na^+^, K^+^, and Ca^2+^^10^ and have a central role in the cholinergic anti-inflammatory pathway by maintaining immune homeostasis through the activation and differentiation of immune T cells and reducing pro-inflammatory cytokines.^57^ Moreover, the alpha 7 nicotinic ACh receptor subunit (α7nAChR) regulates blood flow and cardiac hemodynamics^24^ and enhances ACh release.^58^^,^^59^ While mAChRs and nAChRs trigger distinct signaling cascades across various cell types, they operate synergistically. Targeting both mAChRs and nAChRs through VNS or cholinesterase inhibitors improves left ventricular systolic function, prevents progressive left ventricular enlargement, and modulates the inflammatory response.^52^^,^^60^ The following sub-sections outline the cardioprotective effects of ACh in mitigating CVD through actions on mAChRs and nAChRs.

### mAChRs

Type 2 muscarinic ACh receptors (M_2_AChRs) are the most commonly present receptor in the mammalian heart that activates several cardioprotective signaling pathways.^61^ Previous studies have shown that elevated ACh secretion in an injured heart promotes cardiomyocyte proliferation, suppresses ROS production, and prevents heart injury exacerbation via M_2_AChR.^62^^,^^63^^,^^64^^,^^65^ Activation of M_2_AChR also reduces mitochondrial oxidative damage in a DOX-induced rat model. This effect is mediated by Synapsin I, leading to heightened mitochondrial dynamics, while concurrently mitigating sympathetic activity and diminishing cardiac cell death, and necroptosis.^16^^,^^66^ ACh prevents cell apoptosis by inhibiting the action of angiotensin II (Ang II) and inhibits ROS production as well as cardiac hypertrophy by the activation of sirtuin 3/AMP-activated protein kinase (SIRT3-AMPK) signaling.^67^^,^^68^^,^^69^ ACh also prevents the progression of HF and cardiac remodeling through its inhibition of Ang II, improving survival rates in HF animal models, including mice, rats, and canines.^9^^,^^39^^,^^70^^,^^71^ Furthermore, ACh has the ability to activate superoxide dismutase, a crucial ROS-detoxifying enzyme in mitochondria and cytoplasm, which suppresses ROS production and protects against oxidative stress in I/R.^72^^,^^73^

Several studies have indicated that an increase in ACh levels activates cell survival mechanisms in the heart against I/R.^74^ ACh protects cardiomyocytes from ischemia through the transcription factor HIF-1α and downstream gene expression for cell survival. However, further studies need to be performed to fully identify these signaling pathways.^75^^,^^76^ Additionally, type 3 muscarinic ACh receptor (M_3_AChR) is found abundantly in cardiac fibroblast and plays a critical role in fibroblast proliferation. The activation of M_3_AChR during cardiac fibrosis leads to the inhibition of the mitogen-activated protein kinase (MAPK) signaling pathway, including p38MAPK and ERK1/2 associated with reduced collagen production and cardiac fibrosis.^77^

### nAChRs

In the injured heart, ACh also binds to α7nAChR on various cell types, including cardiomyocytes, monocytes, macrophages, endothelial cells, and others involved in immune responses,^10^ thereby regulating systemic inflammatory responses in CVD patients.^78^ ACh inhibits the production of inflammatory cytokines such as tumor necrosis factor (TNF), interleukin (IL)-6, IL-1β, and IL-18, which are responsible for activating T cells.^4^^,^^21^^,^^66^^,^^79^^,^^80^ Additionally, ACh stimulates macrophages through paracrine signaling, resulting in a reduction in the release of pro-inflammatory cytokines, the uptake of oxidized LDL, and the buildup of cholesterol within macrophages.^10^^,^^81^

Through α7nAChR, ACh attenuates endothelial dysfunction and promotes vasodilation, improving blood flow to the injured heart.^66^ Li et al.^81^ showed that vascular injury in α7nAChR knockout mice led to vascular remodeling, arterial inflammation, and chemokines induction and increased oxidative vascular stress. Activation of α7nAChR can enhance tissue repair as well as reduce cardiac fibrosis damage.^10^^,^^82^ As cardiac fibrosis originates from endothelial cells through a process known as endothelial-to-mesenchymal transition,^83^ α7nAChR has the ability to significantly inhibit IL-1β-induced endothelial-to-mesenchymal transition, nuclear factor κB, and the induction of autophagy leading to the attenuation of cardiac fibrosis.^13^^,^^76^

In addition, ACh plays a significant role in cardiac angiogenesis via α7nAChR during cardiac hypertrophy and MI. Elevating ACh levels in MI mice enhances angiogenesis through vascular endothelial growth factor, leading to increased coronary arterial wall thickness and tube formation.^68^^,^^84^ Moreover, α7nAChR activation also plays a critical role in attenuating ROS by promoting mitochondrial fusion via upregulation of mitofusins (1–2) and thus inhibiting DOX-induced autophagy.^16^

The cardioprotective function of ACh is intricate, involving various downstream pathways mediated by mAChRs and nAChRs to prevent cell death and avert cardiac dysfunction in the injured human heart. Thus, further research is required to identify and evaluate therapeutic approaches aimed at increasing ACh levels and enhancing the activation of mAChRs and nAChRs without adverse effects.

## Challenges and constraints to translate current strategies to clinical studies and alternative approaches

ACh has exhibited protective effects in various *in vivo* animal myocardial damage models. However, these benefits have been poorly translated into clinical trials. The primary challenge in translating ACh’s attributes to clinical trials is its rapid hydrolysis in the presence of cholinesterase and the widespread distribution of ACh receptors throughout the body. Moreover, in 1985, Shepherd and Vanhoutte^85^ found that ACh can detect coronary artery spasms (CADs). The current method for identifying CAD in patients is through intracoronary injection of ACh (ACh-provocation test). This technique is highly sensitive and specific for detecting various types of CAD, including epicardial coronary spasm, microvascular spasm, microvascular dysfunction, and coronary stenosis.^86^^,^^87^ Abnormal vascular responses to ACh, such as ischemic electrocardiogram changes, may be a consequence of endothelial dysfunction and hyperconstriction of vascular smooth muscle. The ACh-provocation test holds potential for tailoring treatment for CAD patients, as it is considered safe with irreversible non-fatal complications. However, it is crucial to note that injecting large doses of ACh (>100 μg) into the right coronary artery of CAD patients may lead to bradycardia lasting up to 45 min^88^^,^^89^

Several methods are commonly employed to elevate ACh levels in the neuronal cholinergic system, such as VNS via implanted stimulators or electrical stimulation and vagal efferent/afferent stimulation. Additionally, ACh levels can be increased in both the neuronal cholinergic system and NNCS through pharmacological interventions, including cholinesterase inhibitors. This section discusses current pre-clinical and clinical studies aimed at elevating ACh levels using VNS and cholinesterase inhibitors, examining their limitations and constraints, including the invasiveness of VNS and the adverse drug reactions (ADRs) associated with cholinesterase inhibitors. Furthermore, we explore potential alternative strategies to enhance ACh levels in the heart, including stem cell therapy, gene therapy, microRNA (miRNA) therapy, and the use of nanoparticles, highlighting the need for more targeted and less invasive approaches.

### VNS

VNS is frequently used to elevate myocardial ACh levels within the neuronal cholinergic system, suggesting its potential as a therapeutic strategy to restore the autonomic balance and its anti-inflammatory effect.^90^ Numerous studies have demonstrated that VNS reduces infarct size, improves ventricular function, attenuates ROS production, and decreases ventricular fibrillation in I/R animal models,^74^^,^^75^^,^^91^ in hypotension and HF models,^92^ as well for a DOX-induced rat model^93^^,^^94^ through mAChRs and nAChRs. Uitterdijk et al.^95^ demonstrated that stimulating the vagus nerve in an ischemic swine model 5 min before reperfusion and continuing for 15 min post-reperfusion resulted in a significant reduction in infarct size. This intervention also led to a decrease in macrophages and neutrophils within the infarct area and mitigation of the no-reflow phenomenon through nAChRs.^96^^,^^97^ VNS exhibits protective effects against cardiomyocyte necrosis and microvascular obstruction, which could prevent reperfusion injury through both muscarinic and nicotinic pathways.^65^^,^^98^ Furthermore, Li et al.^99^ demonstrated that 6 weeks of VNS in HF mice prevented long-term remodeling and improved overall survival.

Despite the evident promise of VNS as a therapeutic strategy against I/R, Buchholz et al.^23^ showed that continuous VNS for 10 min before ischemia significantly increased the infarct size in a rabbit MI heart model. This opposite effect to what is reported earlier could be due to differences in VNS protocols and model species.^100^ Moreover, implanting electrodes to stimulate VNS is invasive, and the optimal electrical dosage remains uncertain.^101^ For instance, Sun et al.^102^ initially employed an electrical voltage of 10 Hz for 0.5 ms, which was subsequently fine-tuned to achieve a 10% reduction in heart rate in rats. This approach significantly constrained infarct size through the mAChRs pathway, mitigating cardiac dysfunction via the nAChRs pathway and extending their survival. While Shao et al.^103^ used a pulse frequency of 4 Hz and an intensity of 6 V to stimulate both right and left vagus nerves, ameliorating the myocardial function in rats by decreasing TNF-α level and arrhythmia score and increasing the expression of α7nAChR. Xue et al.^104^ used electrical voltage from 2 to 4 V to achieve a 10% decrease in the baseline heart rate. This method regulated metabolic homeostasis, modulated mitochondrial function and endoplasmic reticulum stress, and prevented myocardial necrosis and contractile dysfunction during MI in rats. This indicates that the effectiveness of VNS is significantly influenced by factors such as the electrical voltage pulses, stimulation duration, timing, and electrode positioning. These findings underscore the importance of carefully considering these parameters in pre-clinical trials to aim at safeguarding the myocardium against cardiac injury.

Among existing clinical trials, the first pilot study of VNS using CardioFit implantable system showed that VNS may improve quality of life and left ventricle function in chronic HF patients.^105^ However, large cohort clinical studies have shown mixed results.^106^ For instance, the INOVATE-HF clinical trial evaluated the impact of VNS in patients with HF, involving 390 individuals who received implants. Within 90 days, 46 complications occurred in 37 patients, and, after 16 months, VNS failed to demonstrate efficacy in reducing the mortality rate or HF-related events. Furthermore, it did not induce reverse remodeling or increase the left ventricle ejection fraction in this cohort.^107^ The ANTHEM-HF study investigated the impact of continuous cyclic stimulation on both the left and right vagus nerves in HF patients. One patient passed away 3 days after experiencing an embolic stroke during implantation while, in the remaining 59 patients, the devices were successfully implanted. At the six-month mark, the patients showed improvements in left ventricular ejection fraction, left ventricular end-systolic diameter, time-domain of heart rate variability, and high-sensitivity C-reactive protein levels. These findings indicate potential autonomic and anti-inflammatory effects of the treatment.^108^ Hence, it is crucial to acknowledge that VNS is an invasive procedure, and uncertainties persist regarding its safety and potential effects on the human heart.

Despite the fact that VNS is Food and Drug Administration approved for pharmaco-resistant depression, epilepsy, and stroke rehabilitation, the device implantation involves perioperative risks. Potential adverse effects include bradyarrhythmias, development of peritracheal hematoma, dyspnea, and respiratory complications.^109^^,^^110^ Furthermore, the establishment of an appropriate protocol for VNS in ischemic and HF patients remains unresolved, necessitating optimal techniques, and future studies are needed to discern the impact of long-term VNS on acute and chronic myocardial damage.^3^ Given the invasive nature of VNS and the perioperative risks associated with device implantation, alternative methods to modulate the cholinergic system must be considered. For instance, the application of transcutaneous VNS (tVNS) could provide the best clinical outcome and overcome surgical VNS limitations.^111^ The tVNS device is placed on either the anterior wall of the outer ear canal (tragus) or the cymba conchae, and it is inexpensive, low risk, and easy to administer. For instance, Choudhary et al.^101^ demonstrated that tVNS improves cardiac function and reduces MI area in rats. A 6-months clinical study from Stavrakis et al.^112^ demonstrated that the tVNS device reduces atrial fibrillation burden and TNF-α level and suppressed inflammation in patients with paroxysmal atrial fibrillation. However, there is a relative paucity of literature surrounding tVNS operation, functionality, and therapeutic effects.^113^ Future research is needed to identify the most efficient and safest approaches to enhance ACh levels in the heart and reduce side effects to the patients.

### Cholinesterase inhibitors

A non-invasive pharmacological treatment could serve as a potential alternative to chronic VNS therapy, offering a means to target both the neuronal cholinergic system and NNCS. Donepezil, rivastigmine, pyridostigmine, and galantamine are reversible noncompetitive cholinesterase inhibitors. Donepezil is commonly used to prevent progressive neuronal damage,^114^^,^^115^ improve cognitive function, and delay the progression of Alzheimer’s disease^116^ and vascular dementia.^117^^,^^118^ Clinical trials and meta-analyses suggest that cholinesterase inhibitors, particularly donepezil, may be beneficial in combating CVD due to their anti-inflammatory properties and their ability to increase ACh levels in the heart, as observed in Alzheimer’s disease and dementia patients with CVD.^119^^,^^120^^,^^121^ Ongnok et al.^115^ reported that the administration of donepezil significantly mitigates brain pathologies caused by cardiac I/R. This treatment led to increase of BBB junction proteins expression, reduced brain inflammation and oxidative stress, improved mitochondrial function and dynamics, and alleviated amyloid-β accumulation and microglial activation. Furthermore, the cohort study by Nordström et al.^122^ indicated that the use of cholinesterase inhibitors was associated with a 35% reduced risk of MI and death in 7,073 individuals diagnosed with Alzheimer’s disease. However, the study was observational, and the patients had no prior history of CVD. Additionally, healthier patients received higher doses of cholinesterase inhibitors, which was more effective than the standard dose.^123^ Another cohort study found that dementia patients receiving cholinesterase inhibitors have a significantly lower risk of acute coronary syndrome. Consequently, donepezil may protect endothelial cells and improve cardiac vagal activity.^120^

Donepezil has demonstrated favorable cardioprotective effects in animal models against DOX-induced cardiotoxicity,^16^^,^^66^ MI,^124^ I/R,^125^ and HF.^17^ Additionally, Kakinuma et al. demonstrated that donepezil acts as an amplifier of NNCS activity by enhancing ACh synthesis through upregulating ChAT promoter activity in cardiomyocytes^126^ and cholinergic nerve cells.^35^ This could indicate that donepezil increases mAChRs and nAChRs activity by preventing ACh degradation and providing cardioprotective effects against CVD. Despite the impact of cholinesterase inhibitors on CVD, the benefit-to-harm ratio remains a crucial issue for clinical trials and adverse effects of cholinesterase inhibitors are significant. An analysis by Kröger et al.^127^ showed that the ADRs are neuropsychiatric (31.4%), gastrointestinal (15.9%), and cardiovascular (11.7%) disorders found in 18,955 reports (out of 43,753 ADRs). Cardiovascular disorders include a significant increase in the risk of bradycardia,^128^ hypotension,^129^^,^^130^ cardiac arrhythmia,^131^^,^^132^ and syncope.^133^ While a recent meta-analysis study found no association between donepezil and those cardiovascular disorders,^134^ donepezil still leads to ADRs such as tiredness, panic, sweating, diarrhea, vomiting, muscle tension, speech difficulty, and involuntary tremors.^135^ Hence, further research is needed to clarify the protective effects and ADRs of cholinesterase inhibitors against CVD.

### Alternative approaches to target the cholinergic system

**Figure 2:**
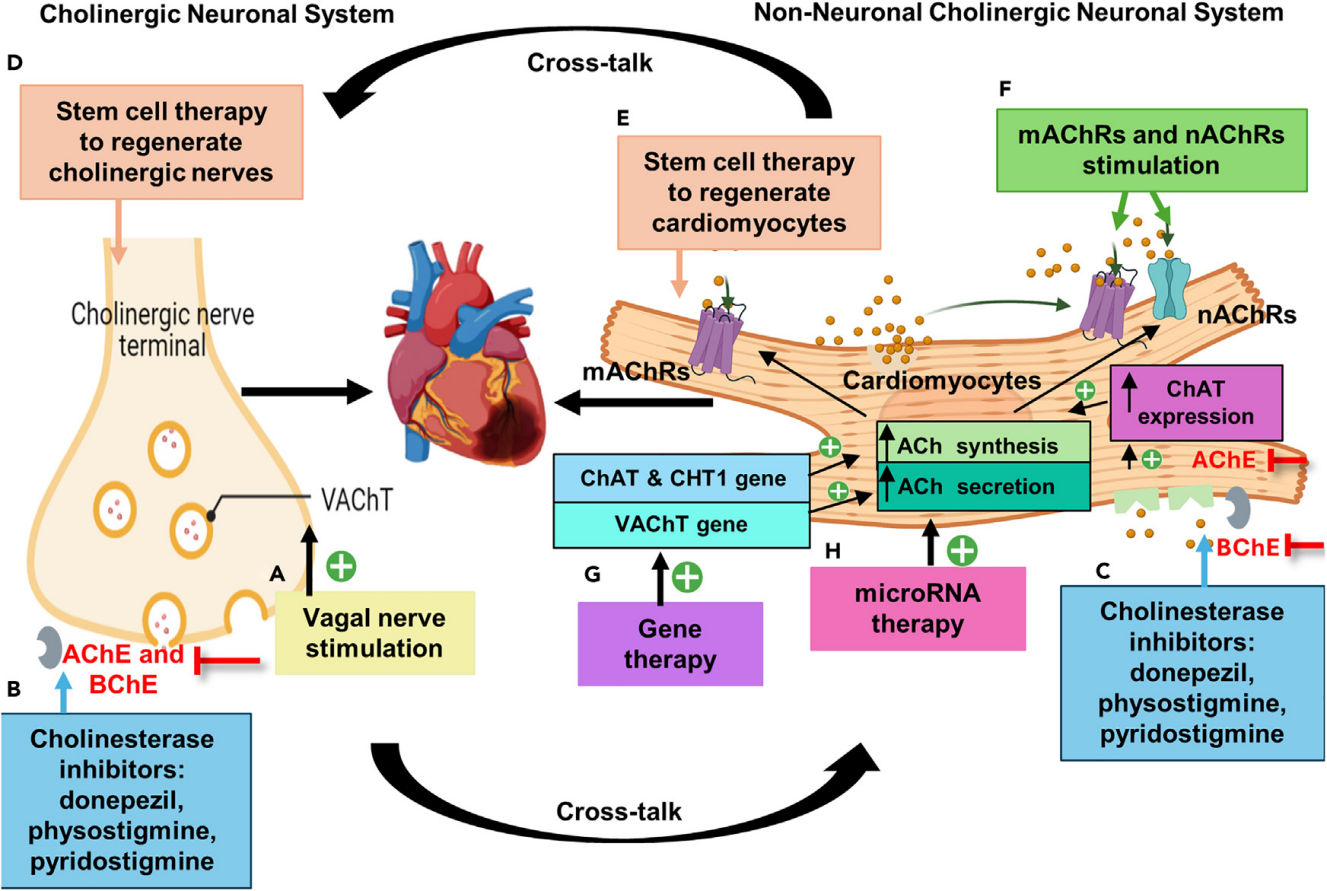
Schematic illustration of therapeutic approaches to target and elevate ACh levels in the infarcted heart (A) These include vagal/vagus nerve stimulation to produce and secrete ACh. (B and C) The other approach is through cholinesterase inhibitors to attenuate the degradation of ACh and prolong ACh effect in the extracellular space. Donepezil can simultaneously inhibit cholinesterase enzymes and increase ChAT expression. (D and E) An alternative approach would be the use of stem cell therapies to either repopulate cholinergic neurons or replace damaged cardiomyocytes respectively. (F) A fourth approach could include a positive allosteric modulator to stimulate mAChRs and nAChRs activity. (G) Gene therapy could be a therapeutic strategy to increase the expression of the cholinergic genes such as ChAT, CHT1, and VAChT, which are responsible for ACh synthesis and secretion. (H) Another possibility is targeting the cardiac miRNAs by diminishing levels of miRNAs that promote ACh degradation. ACh, acetylcholine; AChE, acetylcholinesterase; BChE, butyrylcholinesterase; ChAT, choline acetyltransferase; CHT1, high-affinity choline transporter; mAChRs, muscarinic acetylcholine receptors; nAChRs, nicotinic acetylcholine receptors; VAChT, vesicular acetylcholine transporter.

Multiple pieces of evidence strongly support the cardioprotective effects of ACh against CVD. Nevertheless, therapeutic approaches to target the cholinergic system remain uncertain and limited, warranting further exploration and investigation. This section proposes alternative approaches for future research, including ACh receptor stimulation, stem cell therapy, gene therapy, miRNA therapy, and nanoparticle drug delivery (Figure 2). Stem cell therapy offers a promising avenue for replenishing cholinergic neurons and fostering the regeneration of new, functional cardiomyocytes in hearts damaged by disease or injury. This approach could enhance self-repair mechanisms and improve the functionality of the damaged heart.^18^ While replenishing cholinergic neurons through stem cells has not been extensively studied due to the complexity of the human nervous system, cardiac cell-based therapies, such as bone marrow stem cells, embryonic stem cells (ESCs), and induced pluripotent stem cells (IPSCs), have been explored for their potential to regenerate cardiomyocytes that synthesize ACh.^136^^,^^137^^,^^138^ For instance, the transplantation of bone marrow-derived mononuclear cells has been shown to significantly elevate left ventricular ejection fraction in patients with non-ischemic dilated cardiomyopathy and acute MI. However, it has not led to myocardial regeneration.^136^^,^^139^ Moreover, in large phase 2 randomized controlled trials, stem cell therapy yielded modest results, which could be due to the poor quality of transplanting stem cells.^140^^,^^141^ Additionally, there are concerns with ESCs and IPSCs, such as high survival rates potentially leading to teratoma formation.^142^^,^^143^ Despite these challenges, further exploration into stem cell therapy could lead to significant advancements in achieving cardiac regeneration and restoring cardiac function.

Another potential approach could be gene therapy, which aims to deliver the appropriate gene to be expressed and rescue the ischemic heart by increasing ACh synthesis or secretion pathways in cardiomyocytes. However, there are different types of vectors (non-viral and viral vectors) for gene delivery, and the selection of these vectors depends on the pathological condition of CVD patients. Different types of vectors lead to different durations of cardiac expression and transfection efficiency.^144^^,^^145^ Cholinergic markers, including ChAT^42^^,^^46^ or VAChT,^45^ could be considered new targets to increase ACh and restore the balance between the sympathetic and cholinergic systems in the ischemic heart. As mentioned earlier, ChAt-tg and VAChT-tg models provide cardioprotective effects against myocardial damage and ChAt-tg model also acts as a VNS. Moreover, increasing the overexpression of mAChRs and nAChRs could be another approach. Liu et al.^146^ showed that cardiac-specific M3-mAChR-tg mice significantly attenuated the hypertrophic response by reducing the expression of atrial natriuretic peptide and β-myosin heavy chain induced by Ang II. Nevertheless, the selection of the type of vector needs to be thoroughly considered and studied, as the main disadvantage of these vectors is that this technique could induce an inflammatory response.^144^ Therefore, additional research is required to find an effective *in vivo* delivery method, the duration of gene expression in the ischemic heart, and any potential toxicity effects in CVD patients.

miRNAs are essential regulators of gene expression. miRNA therapy could potentially target mRNA to inhibit translation or inhibit the degradation of ACh. For example, Oikawa et al.^147^ demonstrated that the expression of miR-345 was a regulator of the expression of ChAT mRNA in murine hearts. A synthetic inhibitor for miR-345 could be designed to increase ChAT protein expression and ACh synthesis.^18^ While Shaked et al.^148^ demonstrated that AChE-targeting miR-132 regulates inflammatory responses, Hanin and Soreq^149^ identified 116 and 128 microRNA (miRNA/miRs) that target the 3′-untranslated regions of BChE and AChE (47 for synaptic AChE-S variant and 81 for stress-inducible/readthrough AChE-R variant). However, the cholinesterase-targeting miR sequences show no relevance to most of the cholinesterases’ biological functions, suggesting that those sequences have yet to be explored. Hence, further validation is needed to confirm which miRNAs could directly target ChAT, VAChT, and cholinesterases. The effective delivery method and potential toxicity effects of miRNA therapy also remain unclear. More studies are required to find the ideal approach, determine the duration of its effects, and assess its toxicity effects on the heart and other parts of the body. To conclude, targeting the cardiac NNCS through gene or miRNA therapy seems to be the next step.

Furthermore, BChE is abundantly found in the human body,^150^ while AChE is present in various conducting tissues such as nerves, muscles, central and peripheral tissues, and motor and sensory fibers.^22^^,^^151^ BChE and AChE are present in the body anchored by collagen Q and proline-rich membrane anchors.^152^^,^^153^^,^^154^ BChE is also found as a soluble enzyme in the blood, and AChE subunits produce glycosylphosphatidylinositol-anchored dimers and are anchored to red blood cells.^152^^,^^155^^,^^156^^,^^157^ Under normal conditions, AChE has a higher affinity to hydrolyze ACh than BChE; BChE degrades ACh and carboxylic or phosphoric acid ester-containing compounds and plays important pharmacological and toxicological roles.^158^^,^^159^ For instance, increased BChE levels in monkeys^160^ and mice^161^ protected against the toxicity of nerve agents, such as organophosphorus poisoning and cocaine. While AChE inhibition in mice leads to BChE-mediated hydrolysis of ACh,^158^^,^^162^ inhibition of both BChE and AChE could have severe consequences for human health, including lethal intoxication.^162^^,^^163^ Recently, Dingová et al.^164^ showed that AChE anchored to the membrane of neurons and to the extracellular matrix hydrolyzes neuronal-derived ACh while BChE in the extracellular space hydrolyzes cardiomyocytes-derived ACh in mice hearts. Hence, future work should evaluate the distribution of cholinesterases and their role in the human heart prior to finding an optimal cholinesterase inhibitor to increase the ACh level. Therefore, delivering ACh at small doses and targeting the infarcted area using nanoparticles could be a promising therapeutic approach to increasing ACh levels in the heart. This strategy could potentially enhance the therapeutic effects while minimizing systemic side effects, offering a more precise and effective treatment for conditions requiring cholinergic modulation. Various nanocarriers such as dendrimers, nanometals, nanogels, liposomes, nano-emulsions, polymeric nanoparticles, and nanosuspensions have been studied to deliver drugs at small doses. Nanometal and liposomes nanocarriers have proven to be suitable to deliver ACh, a positively charged and hydrophilic molecule. Studies utilizing silver or gold nanoparticles, as well as chitosan nanoparticles, demonstrated neuroprotective effects against alleviating Alzheimer’s disease.^165^^,^^166^^,^^167^ However, these approaches are not currently being explored for CVD. Future research should investigate the potential of these nanocarriers for targeted ACh delivery in CVD, which could open new avenues for therapeutic interventions.

## Conclusions and future directions

While inhibition of the cholinergic system leads to an increased heart rate, loss of cardiomyocytes,^2^ and increase of production of ROS,^168^ there is evidence that the physiological role of both the neuronal cholinergic system and NNCSs protects the heart against ischemic and chronic myocardial damage.^3^^,^^14^^,^^19^^,^^48^ For instance, the cholinergic system prevents cell apoptosis, inhibits ROS production and cardiac hypertrophy,^61^^,^^62^^,^^63^ and prevents the progression of HF and cardiac remodeling.^9^^,^^38^^,^^64^^,^^65^ Moreover, the cholinergic system provides beneficial immunomodulatory effects following heart tissue injury, such as stimulating macrophages, inhibiting the release of pro-inflammatory cytokines,^10^^,^^75^ attenuating endothelial dysfunction,^60^ and activating cardiac angiogenesis.^68^ However, the relationship between the neuronal cholinergic system and NNCS remains to be fully understood. Understanding the precise mechanisms and interactions between the neuronal cholinergic system and NNCS will also be vital for designing effective treatments.

Additionally, excessive production of ACh and its synthesis can lead to toxicity and bradycardia. Therefore, it is crucial to identify optimal methods for enhancing ACh levels in the heart. VNS and cholinesterase inhibitors are commonly employed to elevate ACh levels in the heart in pre-clinical and clinical studies. However, VNS is invasive, and cholinesterase inhibitors can cause ADRs. Hence, future research should focus on developing targeted and controlled delivery systems to increase ACh in cardiac tissues without causing adverse effects. This includes exploring the use of stem cell therapy, gene therapy, miRNA therapy, and nanoparticles to support and enhance the cholinergic system’s protective functions in the heart. In Figure 2, we also mention the potential use of positive allosteric modulators to selectively target specific subtypes of mAChRs and/or nAChRs, potentially increasing receptor affinity for ACh.^169^ However, current positive allosteric modulators lack specificity and often interact with multiple subtypes, leading to major ADRs such as increased heart rate and drowsiness due to the varied functions of each subtype and subunit of ACh receptors.^170^^,^^171^ Future research should focus on developing more targeted approaches for increasing ACh levels in the heart. This could involve the use of nanoparticle delivery systems or gene therapy to selectively enhance ACh production in cardiac tissue.

More importantly, it is crucial to understand the systemic interactions between the cardiac cholinergic system and other organs, as this will help elucidate the broader physiological impacts of modulating the cholinergic system in the heart. Moreover, long-term studies are necessary to evaluate the safety and efficacy of these alternatives approaches and the long-term effects and safety of enhancing ACh levels in the heart, including the chronic impacts on cardiac function and potential off-target effects. Exploring the potential benefits of combining cholinergic modulation with other therapeutic approaches, such as anti-inflammatory treatments or antioxidants, could provide a more comprehensive protective strategy against heart diseases. Addressing these future directions will help harness the full therapeutic potential of the cholinergic system for cardiovascular health and pave the way for innovative treatments for CVD.

### Limitations of the study

In this review, we summarized the protective role of the cholinergic system, which produces and releases ACh during myocardial damage. We also highlighted the challenges and limitations of current approaches and suggested alternative approaches to elevate the ACh level in the injured heart. Although our review may not include all relevant studies due to limitations in the literature search, it offers a general overview. Certain findings, particularly ambiguous ones, might not be adequately introduced or discussed. We recommend more in-depth research and discussions to better clarify the roles of ACh in CVDs.

## Acknowledgments

C.G. was supported by a University of Sydney Kick-Start grant, a Cardiothoracic Surgery Research Grant, UTS Seed Funding, 10.13039/501100022907Catholic Archdiocese of Sydney Grant for Adult Stem Cell Research, Perpetual IMPACT, 10.13039/501100001208Heart Research Australia grant and Heart Research Institute Fellowship. X.W. was supported by a National Heart Foundation Future Leader Fellowship and a Baker Fellowship. C.L.C.M. was supported by a NSW Waratah Scholarship.

## Author contributions

C.L.C.M. and C.G. had the idea for the review. C.L.C.M. performed the literature search and drafted the original manuscript. X.W. and C.G. critically revised the work.

## Declaration of interests

The authors declare no competing interests.
